# Supplementary material for: Identification of photoperiod-induced specific miRNAs in the adrenal glands of Sunite sheep (Ovis aries)
Source: Front Vet Sci. 2022 Jul 22;9:888207. doi: 10.3389/fvets.2022.888207 (PMC9354845; doi:10.3389/fvets.2022.888207)
Supplement: Supplementary file 1 [file Data_Sheet_1.ZIP › Supplementary file/miRDeep2 file.zip!/miRDeep2 file/Novel_Structure/SPLP42A3/Novel_184.pdf]

Provisional ID	:	NC_019464.2_15720
Score total	:	2
Score for star read(s)	:	-1.3
Score for read counts	:	0
Score for mfe	:	1.7
Score for randfold	:	1.6
Score for cons. seed	:	
Total read count	:	120
Mature read count	:	120
Loop read count	:	0
Star read count	:	0

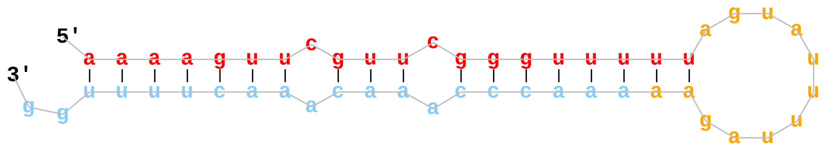

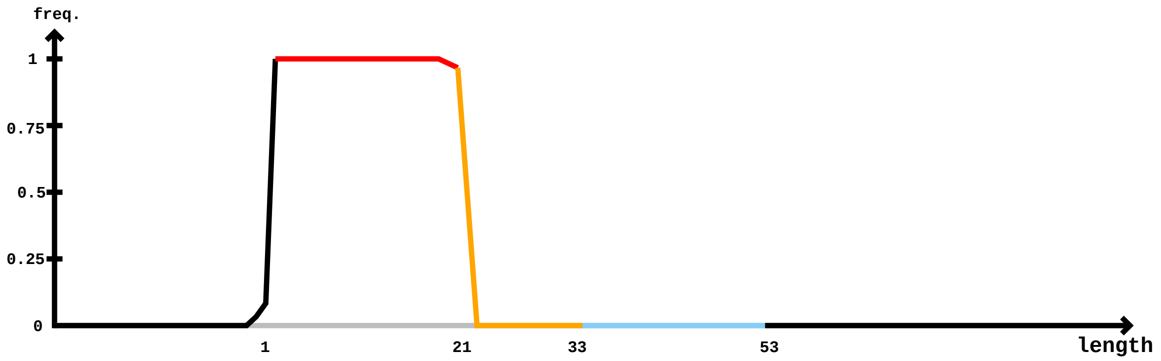

Mature Star

```
5'- gggaauauauuagguuggcccaaaaguucguucggguuuuuaguauuuuagaaaaacccaaacaaacuuuugggcaaacccgauauuucuuaaaguccuuuucuuugugg   -3'    exp
    .((((.(((((.((((.(((((((((((.(((.((((((((..........)))))))).))).))))))))))).)))).)))))))))(((((.......)))))...    reads      mm         sample
    ...................cAaaaaguucguucggguuuu......................................................................    1          1          seq
    ...................cAaaaaguucguucggguuuuu.....................................................................    3          1          seq
    ....................caaaaguucguucggguuuu......................................................................    1          0          seq
    ....................Aaaaaguucguucggguuuuu.....................................................................    5          1          seq
    .....................aaaaguucguucggguuu........................................................................    2          0          seq
    .....................aaaaguucguucggguuuuu.....................................................................    103        0          seq
    .....................aaaaguucguuUggguuuuua....................................................................    5          1          seq
```
